# Pacific Northwest birds have shifted their abundances upslope in response to 30 years of warming temperatures

**DOI:** 10.1002/ecy.70193

**Published:** 2025-09-21

**Authors:** Benjamin G. Freeman, Harold N. Eyster, Julian M. Heavyside, Daniel A. Yip, Monica H. Mather, F. Louise Waterhouse

**Affiliations:** ^1^ School of Biological Sciences Georgia Institute of Technology Atlanta Georgia USA; ^2^ The Nature Conservancy in Colorado Boulder Colorado USA; ^3^ Gund Institute for Environment University of Vermont Burlington Vermont USA; ^4^ Department of Zoology University of British Columbia Vancouver British Columbia Canada; ^5^ Canadian Wildlife Service, Environment and Climate Change Canada Delta British Columbia Canada; ^6^ British Columbia Ministry of Water, Lands and Resource Stewardship Nanaimo British Columbia Canada; ^7^ British Columbia Ministry of Forests Coast Area Research Nanaimo British Columbia Canada

**Keywords:** climate change, escalator to extinction, global warming, mountain biodiversity, old‐growth forest, upslope shifts

## Abstract

Mountain species are predicted to respond to warming temperatures by moving to higher elevations that remain relatively cool. Species can track warming by shifting their entire distributions upwards (the “escalator to extinction” hypothesis) or by increasing in abundance in the upper portion of their elevational range while maintaining stable elevational limits (the “upslope lean” hypothesis). Alternatively, mountain species may not change their abundance or distribution despite climate change (the “persist‐in‐place” hypothesis). Here we evaluate these three contrasting hypotheses by analyzing responses of breeding forest bird species to three decades of warming in southwestern British Columbia, Canada. Consistent with the upslope lean hypothesis, species' optimum elevations (elevations of highest abundance) increased by an average of 126 m, approximately tracking upslope movements in temperature isotherms. In contrast, species' elevational range limits were stable on average, contra the escalator to extinction hypothesis. Many individual species had stable distributions and abundances, and species with upslope abundance increases typically maintained stable abundances within the lower elevation portions of their range. Taken together, most species in our study region appear to be responding neutrally or favorably to warming temperatures. Nevertheless, one mountain species, the Canada Jay, Canada's national bird, is declining and vulnerable to the escalator to extinction within our study region. Overall, we emphasize the importance of empirical data—and abundance data in particular—when evaluating mountain species' vulnerability to climate change.

## INTRODUCTION

Mountains are the terrestrial habitat whose biotas are most vulnerable to climate change‐driven extinctions (Urban, 2024). Mountain species are generally predicted to track warming temperatures by shifting their abundances and distributions toward cooler environments, and many mountain species are indeed moving toward higher elevations as temperatures warm (Chen et al., 2011; Lawlor et al., 2024; Lenoir et al., 2020; Steinbauer et al., 2018). Mountain species can track temperature increases via changes in their elevational ranges or in their abundances. The “escalator to extinction” hypothesis proposes mountain species move their lower and upper range limits upslope at similar rates until they eventually run out of room at the mountaintop (e.g., Freeman et al., 2018). In the “upslope lean” hypothesis, mountain species increase in abundance toward the upper end of their distribution while maintaining stable ranges (Breshears et al., 2008; Kelly & Goulden, 2008).

Alternatives to temperature tracking include that mountain species acclimate or adapt to climate change, or that their distributions and abundances are unrelated to temperature (Lenoir & Svenning, 2015). A common consequence of these alternatives is that mountain species maintain stable abundances and distributions as temperatures become warmer (the “persist‐in‐place” hypothesis; this is a null hypothesis of no change). Consistent with this hypothesis, many mountain species have maintained largely stable distributions and abundances despite warming temperatures, particularly in the temperate zone (Freeman et al., 2021; Lenoir et al., 2010; Neate‐Clegg et al., 2024). We motivate our study using the escalator to extinction, upslope lean, and persist‐in‐place hypotheses, but acknowledge additional possible responses to climate change, including in directions opposite to that predicted by climate tracking (Lenoir & Svenning, 2015). One possibility is that these hypotheses represent sequential responses to climate change: initially, mountain species persist‐in‐place, later lean upslope, and then eventually shift to higher elevations.

The escalator to extinction, upslope lean, and persist‐in‐place hypotheses can be tested with survey data documenting changes over time in mountain species' abundances and distributions. However, such studies typically use presence–absence occupancy data and thus cannot assess the upslope lean hypothesis. We highlight the benefit of analyzing abundance data (Lenoir & Svenning, 2015). Abundance data provide direct information on changes in population size associated with changing climate (Gaya & Chandler, 2024; Gilbert et al., 2024), and can identify species whose declines suggest functional extinction, even when the species persists. Moreover, abundance data may serve as an “early warning” system to highlight vulnerable species that might benefit from immediate management interventions. For example, a species that maintains a stable range size might still show strong abundance declines which portend future declines in range (Billman et al., 2024).

Here we examine how breeding birds have changed their abundances and distributions associated with three decades of warming in the temperate old‐growth forest of the Pacific Northwest of North America. We tested contrasting predictions of the escalator to extinction, upslope lean, and persist‐in‐place hypotheses by modeling changes in species abundances across elevations using a flexible approach that incorporates the detection process. Our main finding is that species' optimum elevations (elevations of highest abundance) have shifted upslope at a comparable magnitude to the estimated upslope movement of annual temperature isotherms: this pattern is consistent with the upslope lean hypothesis. Few bird species in our study region appear to be vulnerable to the escalator to extinction, with one notable exception: we find a strong decline in the abundance of the high‐elevation Canada Jay *Perisoreus canadensis*. We argue that a greater focus on measuring abundance changes will help produce management‐relevant data on mountain species' responses to climate change.

## METHODS

### Historical bird surveys

**FIGURE 1:**
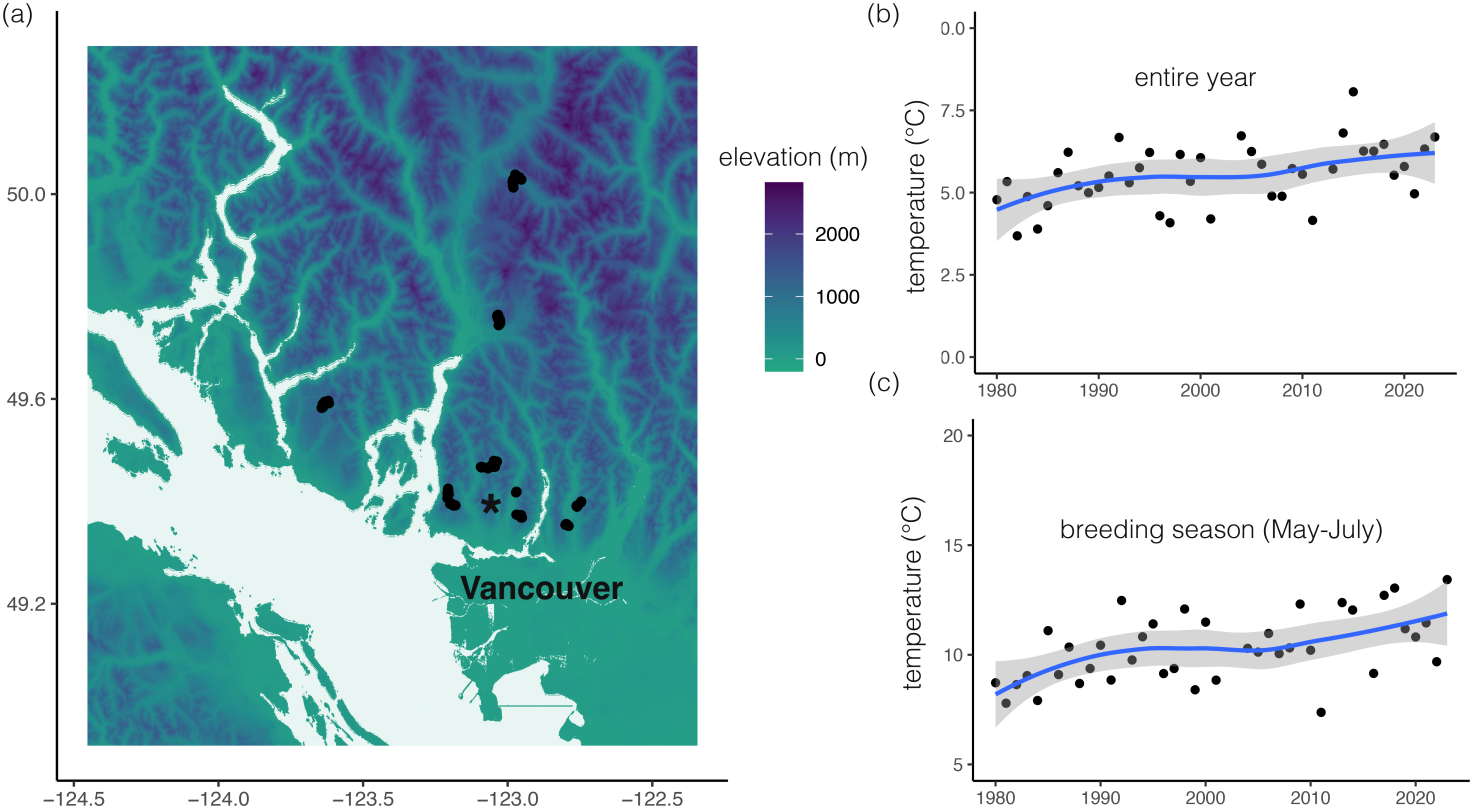
Map of study site in the mountains of southwestern British Columbia, Canada, with point count locations shown as black dots (*N* = 112 point count locations); the Grouse Mountain weather station is shown with an asterisk (a). This region is warming over both the entire year (b) and the bird breeding season (May–July; c); blue trend line and gray band show the locally estimated scatterplot smoothing fit to the data with 95% CI.

We surveyed the breeding bird community of southwestern mainland British Columbia, Canada, just north of the city of Vancouver (Figure 1a). A team led by F. Louise Waterhouse conducted extensive surveys of breeding birds in this region from 1990 to 1993 (Savard et al., 2000; Waterhouse et al., 2002). Here we focus on surveys conducted in old‐growth forest stands, generally defined as old climax forest >250 years old (BC Ministry of Forests and Range and BC Ministry of Environment, 2010). In the 1990s, a team of four surveyors rotated visits to 146 point count stations in old‐growth stands between ~200 and 1440 m; each point count station was located 200 m from the nearest adjacent station, and points were >125 m from edges to sample interior forest conditions. Surveyors worked between dawn and ~9:30 am from May 4 to July 1, avoiding mornings with moderate to heavy rain. Point count stations in old‐growth stands within the Capilano, Coquitlam, and Seymour watersheds were flagged and marked with small placards affixed to trees, and their location marked on stand maps at 1:50,000 scale. Within Cypress, Seymour, Garibaldi, and Tetrahedron Provincial Parks, point count stations were placed along publicly accessible trails, and their location marked on fine‐scale maps. On each visit to a point count station, a surveyor completed a 12‐min, 75‐m radius point count, recording the distance to each detected bird within each of three 4‐min sub‐counts. Individuals detected over 75 m from the point count station were coded as distance = 80 m. Each station was visited an average of 5.33 times (SD = 3.1; ~ 16 4‐min sub‐counts per point count station).

### Modern bird surveys

In February 2023, Benjamin G. Freeman found the publication that described the results of the early 1990s southwestern British Columbia survey while completing a literature scan on systematic surveys of birds along elevational gradients. Benjamin G. Freeman reached out to authors F. Louise Waterhouse and Monica H. Mather, who expressed support for a resurvey and shared key methodological and logistical information. The old‐growth stands originally surveyed all remain unaltered and are embedded within a forested landscape that has remained compositionally similar since the early 1990s—minimal logging has occurred within the study region during this time. We thus assume that habitat for birds in old‐growth stands has remained approximately stable within our study site between historical and modern surveys. Benjamin G. Freeman and Julian M. Heavyside completed point counts between May 30 and June 29, 2023, following the methods used in the original survey, with two deviations: we estimated distances to all detected birds, and further split each 4‐min sub‐count into two 2‐min sub‐sub‐counts. The 2023 resurvey visited 112 point count stations an average of 2 times (standard deviation = 0.23; ~6 4‐min sub‐counts per point count station; we were unable to visit some original point count stations because several roads within watersheds had been decommissioned).

We are confident we placed point count stations in 2023 close to their original location in the early 1990s. At higher elevations, we used trail maps from the original survey to relocate point count stations, and trail paths have not changed in the intervening decades. In the watersheds, we used forest road locations from stand maps to relocate the approximate position of old‐growth stands. Once on site, we relocated old‐growth stands by observing their giant trees and copious large woody debris that were not present in surrounding secondary forests. Within old‐growth stands, we used stand maps to site point count stations. We estimate that modern point count locations were typically within ~50 horizontal meters of their original location, meaning that any error associated with elevation for point count locations between survey periods should be minimal (<10 m of elevation). We have some direct evidence to support our claim of accuracy. Twice in 2023 Benjamin G. Freeman sited point count stations in old‐growth stands within the watersheds using stand maps, then subsequently found placards on tree trunks ~10 m away. F. Louise Waterhouse later informed Benjamin G. Freeman that these placards had marked the location of point count stations in the historical survey, and that all flagging tape and most placards (but clearly not all) indicating the original point count stations were collected upon the completion of the original project in 1993. We recorded the location of all modern point count stations in 2023 using a GPS.

### Climate analysis

We estimated the amount of temperature change that has occurred within our study region using data from the Grouse Mountain weather station (~1100 m) in North Vancouver; most point count locations are within 5–10 km of this weather station. We downloaded daily mean temperature data from 1980 through 2023 from climate‐change.canada.ca/climate‐data in May 2024, then summarized changes in annual mean temperature and in breeding season temperatures (May–July) by fitting linear models to estimate rates of temperature change over time (estimated slopes: annual temperature: 0.032 ± 0.0099, *t* = 3.25, *p* = 0.0023; breeding temperature: 0.061 ± 0.016, *t* = 3.78, *p* = 0.00053). We used these slopes to estimate regional annual temperature changes over the three decades between bird surveys to have been ~1°C (annual temperature) and ~1.8°C (breeding season). A general estimate of lapse rates in mountains is that temperature declines by ~0.6°C for an increase of 100 m in elevation. We therefore estimated that isotherms in our region have shifted upslope by ~166 m (annual temperature) to ~306 m (breeding season temperature). These values are approximate, but nevertheless provide a general expectation for the distribution and abundance changes predicted by the escalator to extinction and upslope lean hypotheses.

### Bird analysis

We conducted analyses in R (R Development Core Team, 2022). We first filtered the point count dataset to account for differences in how historical and modern surveys were conducted. We removed (1) historical surveys conducted before May 23rd, since these may have detected migrants, (2) all detections recorded at a distance greater than 75 m (with the exception of Sooty Grouse, whose booming hoots are highly detectable at long distances), and (3) the point count locations within watersheds that are no longer accessible and hence not visited in 2023. The filtered data contained 112 points sampled during both periods, visited on average 1.9 times in 2023 (SD = 0.28) and 3.6 times (SD = 1.6) in the 1990s.

We then fit Bayesian binomial N‐mixture models—with one model for each of the 56 observed species—to account for nondetection such that:

$$y_{\mathrm{ijk}}\;\sim \text{Binomial}\left(N_{\mathrm{ij}},p\right),$$

where *y*_*ijk*_ is the number of individuals of the species observed in survey period *i* and sampling site *j* and within‐period temporal replicate *k*, *N* is the true abundance after accounting for nondetection, and *p* is the detection probability, treated as constant across time periods for each species (Yamaura et al., 2012). We included two time periods: “historical” (survey data = 1990–1993) and “modern” (survey data = 2023). Due to data limitations for many species, we chose to focus on adding complexity in the models for elevation response, but kept the models simple for the detection probabilities, which did not contain covariates.

We modeled the relationships between each species' abundance and elevation using Huisman–Olff–Fresco (HOF) models (Huisman et al., 1993; Oksanen & Minchin, 2002). These models provide flexibility to fit any unimodal elevation‐response such that:

$$N_{\mathrm{ij}}\sim \text{Poisson}\left(\lambda_{\mathrm{ij}}\right),$$

$$\lambda_{i,j}=M_{i}\frac{1}{1+\exp\left(a_{i}+b_{i}x_{j}\right)}\;\frac{1}{1+\exp\left(c_{i}-d_{i}x_{j}\right)},$$

where *x* is the standardized elevation (Gelman, 2008), and *M, a, b, c*, and *d* are parameters that may vary between survey periods and are fit independently as fixed effects for each period, with one set of parameters for period 1 and one set for period 2. We fit each model in Stan (Carpenter et al., 2017) using Rstan (v. 2.32.3) in R (v. 4.3.2) with four chains, each with 2000 iterations (half used for warm‐up and discarded).

We then used model posteriors to estimate the expected abundance of all species with narrow and well‐defined credible intervals (*n* = 24, see Appendix S1: Figure S1) while accounting for nondetection at each elevation between 201 and 1443 m, the lowest and highest elevation point count locations visited. We quantified species' elevational ranges during each time period by calculating the elevational range within which each species had a median expected abundance >0.2 or where median expected abundance was greater than the 15th percentile of its maximum abundance. We used these historical range limits to classify each species into three categories: broad (living across the entire elevational gradient), low elevation (living at low elevations and with an upper‐elevation range limit), and high elevation (living at high elevations and with a low‐elevation range limit). There were no species that historically lived only in middle elevations with both low‐ and high‐elevation range limits. We assessed changes in abundance within these elevational ranges using overlap between the 89% credible intervals (McElreath, 2018) of each species' abundance at each elevation for the historical and modern periods. If credible intervals overlapped at a particular elevation, we classified the species' abundance as stable at that particular elevation; if the credible intervals for the modern survey period were higher and nonoverlapping, we classified the species' abundance as increasing at that particular elevation, and vice versa for decreasing.

We estimated the optimum elevation of each species during each time period by calculating the elevation at which our models predicted the maximum abundance of a given species during a given time period. We used the uncertainty in our model posteriors to calculate 89% credible intervals associated with the optimum elevation estimates. We then calculated the average change between the historical and modern surveys in optimum elevation across 22 of the 24 species (we excluded Evening Grosbeak *Coccothraustes vespertinus* and Wilson's Warblers *Cardellina pusilla* from this average, since these were absent or nearly absent from one of the time periods).

**FIGURE 2:**
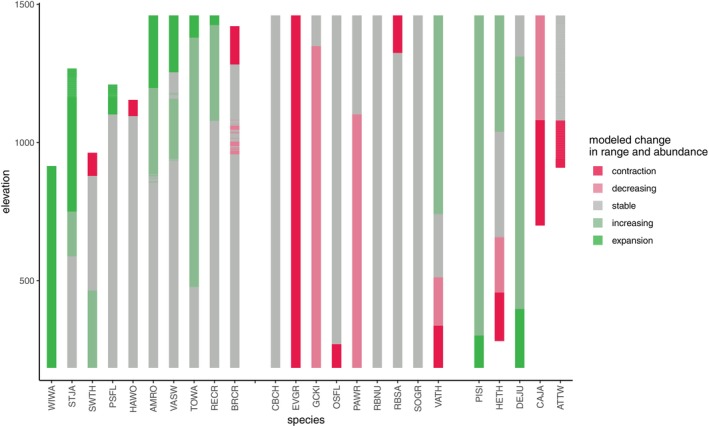
Most species showed changes in their distributions and abundances along the elevational gradient. Columns show 24 common species, organized into three categories based on distributions during the historical survey: Low elevation species (left), species that lived across the entire elevational gradient (middle), and high elevation species (right). Colors show the portion of the elevational gradient where ranges contracted (bright red), abundance decreased (dull red), abundance was stable (gray), abundance increased (dull green), and range expanded (green). We defined species' elevational ranges as the set of elevations where species' expected abundance was greater than 0.1 or greater than 15% of the species' maximum abundance.

We visually inspected individual species' elevational range and abundance changes using the information presented in Figure 2 and Appendix S1: Figures S1–S3 to classify species' overall responses to climate change into six categories: abundance expansion up (consistent with the upslope lean hypothesis), upslope range shift and high‐elevation abundance contraction (consistent with the escalator to extinction hypothesis), stable (consistent with the persist‐in‐place hypothesis), and decline or abundance expansion down (not consistent with any hypothesis).

Last, we used model posteriors to estimate changes in community‐level biodiversity metrics. We estimated total abundance for each elevation in each time period by summing expected abundances (λ in equations above) of all modeled species. We propagated uncertainty from our models into this estimate by calculating the total abundance separately for each elevation using the 4000 model iterations and used these 4000 estimates of total abundance at each elevation to calculate the median and 89% credible intervals (using highest density intervals; HDI) at each elevation. We estimated species richness for each elevation in each time period by summing the number of species our models predicted to occur at each elevation (i.e., *N* in equations above; we drew random draws from Poisson distributions parameterized by the modeled expected abundances (λ), converted integer draws to presence/absence, and then summed across species); we propagated uncertainty analogously to the total abundance estimate. To investigate whether patterns were influenced by irruptive species, we repeated analyses excluding irruptive finch species. All authors have spent significant time living and birding in and around the mountains of southwestern British Columbia. These experiences and intuition about Pacific Northwest birds have likely shaped and informed our hypotheses and interpretations.

## RESULTS

**FIGURE 3:**
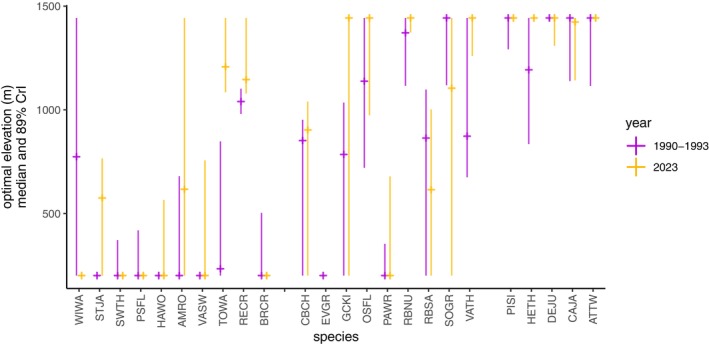
Species' estimated optimum elevations tended to shift to higher elevations between the early 1990s (purple) and 2023 (orange). The organization of this figure follows that of Figure 2; low elevation species are on the left, species with broad elevational ranges are in the middle, and high elevation species are on the right. We calculated optimum elevation in each time period as the elevation of peak modeled abundance, shown in squares; vertical lines illustrate the uncertainty associated with the optimum elevation estimate.

Most species in our study showed changes in elevational ranges or abundances, visualized in Figures 2 and 3 (see also Appendix S1: Figures S1–S3 for detailed species‐specific results). One originally uncommon species over the elevational gradient is now present only at low elevations, where its abundance increased (Wilson's Warbler); another species (Evening Grosbeak, an irruptive finch) was not present at our study site during the modern survey. Of the remaining 22 species, only three resident species showed entirely stable abundances and distributions between the original and modern surveys (Sooty Grouse *Dendragapus fuliginosus*, Chestnut‐backed Chickadee *Poecile rufescens*, and Red‐breasted Nuthatch *Sitta canadensis*; all three live across the entire elevational gradient). Thus, even for birds living within old‐growth forests that are many centuries old, change is the norm over the three decades of our study.

We found no statistical evidence that species have moved their elevational range limits upslope on average. Mean changes in the elevational position of lower and upper range limits were ~30 m upslope, with CIs that overlapped zero (*N* = 22; low‐elevation limit: 32 m; 89% CI = −7 to 71 m; range = −180 to 382; high‐elevation limit = 33 m; 89% CI = −11 to 77 m; range = −106 to 485). In contrast, we found evidence that species' abundances have moved upslope on average: species increased their optimum elevation by an average of 126 m (*N* = 22; 126 m; 89% CI = 28 to 244 m; range = −396 to 792 m; Figure 3). We found similar patterns when analyzing the location of abundance increases or decreases rather than optimums: abundance changes near species' upper elevation limits were nearly all increases, while abundance changes near species' lower elevation limits were a mix of decreases and increases (see Figure 2).

**FIGURE 4:**
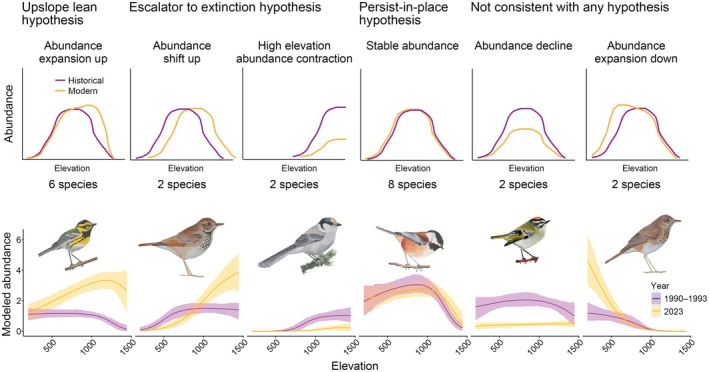
We categorized species' modeled abundance changes into six scenarios and illustrate each scenario with an empirical example. Many species showed upward abundance expansions (6 species), consistent with the upslope lean hypothesis, while some showed abundance shifts up (2 species) or high elevation abundance contractions (2 species), consistent with the escalator to extinction hypotheses. At the same time, many species had stable abundance distributions over time, consistent with the persist‐in‐place hypothesis (8 species). Last, some species showed patterns inconsistent with either hypothesis, either declining across their broad elevational range (2 species), or with downslope abundance expansions (2 species). Example species for each category are Townsend's Warbler, Hermit Thrush, Canada Jay, Chestnut‐backed Chickadee, Golden‐crowned Kinglet, and Swainson's Thrush. Illustrations by H. N. Eyster.

We used modeled abundances from both time points (Appendix S1: Figure S2) to visually categorize individual species into six types of abundance change categories aligned to the motivating hypotheses (Figure 4). Stable abundances and abundance expansions up were the most common responses; these are, respectively, consistent with predictions of the persist‐in‐place hypothesis and the upslope lean hypothesis. We classified species as follows, providing English and Latin names as well as the four‐letter codes used in Figures 2 and 3: Stable abundance = Brown Creeper *Certhia americana* (BRCR), Chestnut‐backed Chickadee *Poecile rufescens* (CBCH), Hairy Woodpecker *Dryobates villosus* (HAWO), Olive‐sided Flycatcher *Contopus cooperi* (OSFL), Pacific‐slope Flycatcher *Empidonax difficilis* (PSFL), Red‐breasted Nuthatch *Sitta canadensis* (RBNU), Red‐breasted Sapsucker *Sphyrapicus ruber* (RBSA) and Sooty Grouse *Dendrogapus fuliginosus* (SOGR); abundance expansion up = American Robin *Turdus migratorius* (AMRO), Pine Siskin *Spinus pinus* (PISI), Townsend's Warbler *Setophaga townsendi* (TOWA), Red Crossbill *Loxia curvirostra* (RECR), Steller's Jay *Cyanositta stelleri* (STJA) and Vaux's Swift *Chaetura vauxi* (VASW); abundance shift up = Hermit Thrush *Catharus guttatus* (HETH) and Varied Thrush *Ixoreus naevius* (VATH); high‐elevation abundance contraction = Canada Jay *Perisoreus canadensis* (CAJA) and American Three‐toed Woodpecker *Picoides dorsalis* (ATTW); abundance decline = Golden‐crowned Kinglet *Regulus satrapa* (GCKI) and Pacific Wren *Troglodytes pacificus* (PAWR); and abundance expansion down = Dark‐eyed Junco *Junco hyemalis* (DEJU) and Swainson's Thrush *Catharus ustulatus* (SWTH). This exercise illustrates the approximate number of individual species in each category; we acknowledge that some species' abundance changes are marginal or could fit multiple categories.

**FIGURE 5:**
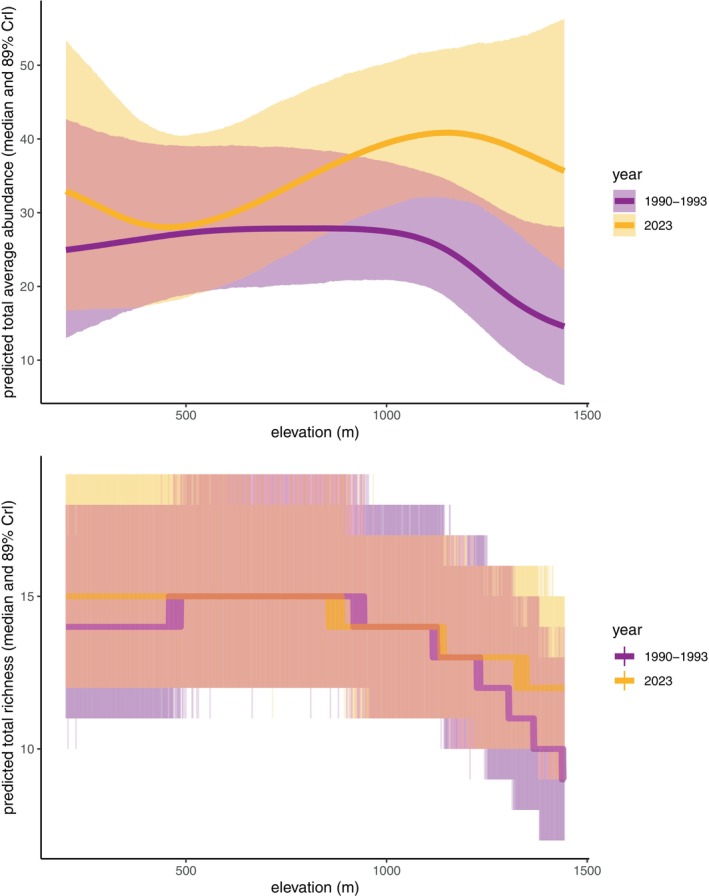
Overall community abundance and species richness were stable at lower elevations but showed slight increases at higher elevations. Trend lines show model predictions; shaded bands show 89% credible intervals. We calculated community abundance as the summed expected abundance across species (a). For species richness, we used the summed predicted occupancy across species, then drew random draws from Poisson distributions parameterized by the modeled expected abundances, converted draws to present/absent, and summed across species to generate the discrete predictions plotted in (b).

At a community level, we found abundance and species richness were stable at lower elevations and increased slightly at higher elevations between time periods (Figure 5, Appendix S1: Figure S4). The increase in modeled abundance at high elevations remained similar when excluding irruptive finches.

## DISCUSSION

We report evidence that species' abundances have moved upslope in Pacific Northwest old‐growth forests associated with three decades of warming temperatures. We surveyed birds living within old‐growth stands embedded in a forested landscape that is predominantly covered with secondary forest. We therefore assume that forest structure within old‐growth stands has been relatively stable during our study period; however, we estimate that annual temperature isotherms have moved upslope by roughly 150 m over this time. The predominant response of the forest bird community to this warming has been to increase in abundance at higher elevations within their ranges. Optimum abundances moved upslope by an average of ~125 m, while changes in lower and upper elevational range limits were minimal. At the community level, we thus find evidence for the upslope lean but not the escalator to extinction hypothesis, with the consequence that the elevational gradient in species richness within our study region has remained largely stable over time (Brodie et al., 2025). When we examined individual species, we found that most species appear to be either persisting in place or exhibiting upslope lean responses, with fewer species showing patterns consistent with the escalator to extinction hypothesis.

Our study adds to evidence that most temperate zone mountain species across the globe are not tightly tracking warming temperatures via upslope range shifts (Freeman et al., 2021). Instead, we find evidence for upslope leans in abundance, as have been reported for forest plants in Europe and North America (Kelly & Goulden, 2008; Lenoir et al., 2008). The lack of upslope shifts in species' range limits may be because temperate mountain species track temperature in time via phenological shifts rather than in space via elevational changes (Neate‐Clegg et al., 2024; Socolar et al., 2017), or because temperate species have broad physiological tolerances that permit acclimation to climate change (Sunday et al., 2012). Regardless, the overall pattern of limited observations of climate tracking via upslope range limit changes in temperate zone species (e.g., Sanczuk et al., 2024; Zillig et al., 2024) is at odds with the assumption of tight climate tracking used by many models when forecasting species' long‐term responses to warming temperatures. This discrepancy suggests temperate mountain species may be less vulnerable to climate change‐driven extinction than is often assumed (see also Steinbauer et al., 2018), at least over shorter time periods.

In contrast, species for which climate has direct fitness consequences may show tight climate tracking. For example, previous research documented that Canada Jay populations in eastern Canada are sensitive to climate because milder winters cause food caches to rot, decreasing winter survival (Sutton et al., 2019). We found that Canada Jays are declining in abundance and shrinking in elevational range in southwestern British Columbia, though we do not have similar data linking climate to fitness from our study region. The case example of the Canada Jay underscores how detailed mechanistic information on how climate influences range limits may prove useful when predicting species' climate change vulnerability (Alexander et al., 2015; Brown & Vellend, 2014; Riddell et al., 2019). Our results affirm that climate change is likely to have negative impacts on some Pacific Northwest bird species such as the Canada Jay, but suggest that habitat loss and other threats are of greater importance for most species, at least over decadal time scales.

### The value of estimating abundance changes

We highlight the power of modeling abundance changes to infer responses to climate change. If we had only analyzed changes in presence–absence data, we would have concluded that little change had occurred between our survey periods. Instead, our analysis of abundances revealed marked changes in elevational patterns of abundance for many species: many species' optimum abundances moved upslope, approximately following expectations given upslope shifts in annual temperature isotherms. Abundance is a more fine‐grained measure of species' responses to environmental changes than presence–absence, and provides direct information on population changes that may be relevant to land managers (Gaya & Chandler, 2024). Many species showed dramatic abundance changes despite relatively stable elevational ranges: for example, Varied Thrush and Townsend's Warbler increased in abundance by ~3× at high elevations, while Golden‐crowned Kinglet and Pacific Wren declined by ~4× throughout much of their range. Abundance changes may be an early indicator of subsequent distributional changes, with abundance declines preceding range contractions or abundance increases preceding range expansions (Billman et al., 2024). For example, Canada Jay declined in abundance in the lower portion of its elevational range and contracted at its lower elevation range limit, while Dark‐eyed Junco increased in abundance in the lower portion of its elevational range and expanded at its lower elevation range limit.

To help promote analyses of abundance changes along environmental gradients, we developed a new methodology using Huisman–Olff–Fresco models to fit flexible response curves modeling abundance in each time period (Huisman et al., 1993; Oksanen & Minchin, 2002). These models fit a variety of responses along gradients, allowing for modeling abundance distributions that are not well described by linear or quadratic responses. These models have been previously used to estimate range changes along elevational gradients (e.g., Lenoir, 2009). Here we developed a method to incorporate non‐detection in Huisman–Olff–Fresco models, and provide example code and documentation, allowing for these methods to be used for a broader range of taxa and study designs.

### Implications for forest conservation and management

Our findings provide information to support climate change informed planning and management within our study region. The original study we used as a pre‐warming baseline recommended retaining old‐growth forest across elevations, as lowland and mountain old‐growth stands harbor different bird communities (Waterhouse et al., 2002). Here we repeat this recommendation, as both lowland and mountain stands have maintained or increased in forest bird species richness and abundance. We found that most lowland species continue to live in lowland forests with stable abundances. Hence, lowland old‐growth forests in our study region retain their conservation value for birds as temperatures warm. One possible reason is that mature forests have unique microclimates that buffer temperature fluctuations and maintain stable microclimates, potentially allowing mature forests to act as microrefugia for forest‐dwelling species even as macroclimate changes (Betts et al., 2018; Davis et al., 2019; Kim et al., 2022; Lenoir et al., 2017). At the same time, most species shifted their optimum elevations upslope, and mountain forests thus show slight increases in abundance and species richness. We interpret this to mean that mountain old‐growth forests in our study region have stable or slightly increasing conservation value to birds. Data from other taxa are desirable to generalize beyond the forest bird community: a previous study from the Pacific Northwest mountains also reported minimal changes in the elevational distributions of plants within undisturbed areas (Wilson et al., 2024).

Our abundance analyses highlight a handful of species with marked increases or decreases in abundance. Most species had stable or increasing abundance overall and thus appear to respond favorably or neutrally to climate change within old‐growth stands. Yet some species showed general steep declines: for example, Pacific Wren and Golden‐crowned Kinglet declined sharply in abundance throughout their broad elevational ranges. These species are still present in old‐growth forests throughout the gradient but are no longer abundant: ongoing monitoring will be important to determine further population changes and to test the hypothesis that abundance declines precede range contractions. We particularly highlight a decline in the abundance of Canada Jay, the national bird of Canada. Canada Jays were common at higher elevations in our study region in the early 1990s, but we detected very few in the modern survey. We have no evidence that Canada Jays have shifted to still‐higher elevations, as we did not detect Canada Jays while conducting surveys in June 2023 at elevations >1400 m that were not part of the original survey (and hence not included in analyses presented in this manuscript). It is perhaps noteworthy that Steller's Jay, a lower elevation species, has increased in abundance at middle elevations in our study region: future studies could investigate whether competition between these two corvids plays a role in the Canada Jay's observed decline.

### Recommendations for future work

Our snapshot surveys show marked changes in forest bird communities associated with climate change, even within old‐growth stands. Continued monitoring can evaluate ongoing changes, while fieldwork can test potential mechanisms by which climate directly or indirectly drives observed changes in bird abundance and distribution. In particular, expanding the scope of surveys in this region to include a range of forest seral stages and landscape conditions could identify how abundances and distributions are changing across the landscape (Eyster et al., 2024; Leupin et al., 2011). This would help distinguish changes in bird populations due to climate from those due to anthropogenic or natural disturbances to inform trajectories for ecosystem recovery and restoration. For example, one hypothesis is that changes in bird abundances are related to changes in forest structure that influence microclimate (Kim et al., 2022); this could be tested by collecting modern forest structure data to compare to that collected during the original study (Waterhouse et al., 2002). By repeated sampling across forest types, it will be possible to assess the changes we report here for forest birds in this study region at landscape scale. Overall, our results show that most species' ranges are not changing as predicted by simple climate tracking models (Langham et al., 2015), highlighting the importance of empirical data to determine mountain species' vulnerability to climate change.

## AUTHOR CONTRIBUTIONS

Benjamin G. Freeman conceived the project with input from F. Louise Waterhouse. F. Louise Waterhouse and Monica H. Mather led the historical survey; Benjamin G. Freeman and Julian M. Heavyside conducted the modern survey. Harold N. Eyster led the analysis with input from Benjamin G. Freeman and Daniel A. Yip. Benjamin G. Freeman wrote the first draft, and all authors assisted with revisions.

## CONFLICT OF INTEREST STATEMENT

The authors declare no conflicts of interest.

## Supporting information


**Data S1.** Supporting information.

## Data Availability

Data and code (Freeman et al., 2025) are available in Zenodo at https://doi.org/10.5281/zenodo.16049071.
