# Supplementary material for: Pacific Northwest birds have shifted their abundances upslope in response to 30 years of warming temperatures
Source: Ecology. 2025 Sep 21;106(9):e70193. doi: 10.1002/ecy.70193 (PMC12450588; doi:10.1002/ecy.70193)
Supplement: Supplementary file 1 — Data S1. Supporting information. [file ECY-106-e70193-s001.pdf]

Appendix S1

Pacific Northwest birds have shifted their abundances upslope in response to 30 years of warming temperatures

Benjamin G. Freeman, Harold N. Eyster, Julian M. Heavyside, Daniel A. Yip, Monica H. Mather & F. Louise Waterhouse

Ecology

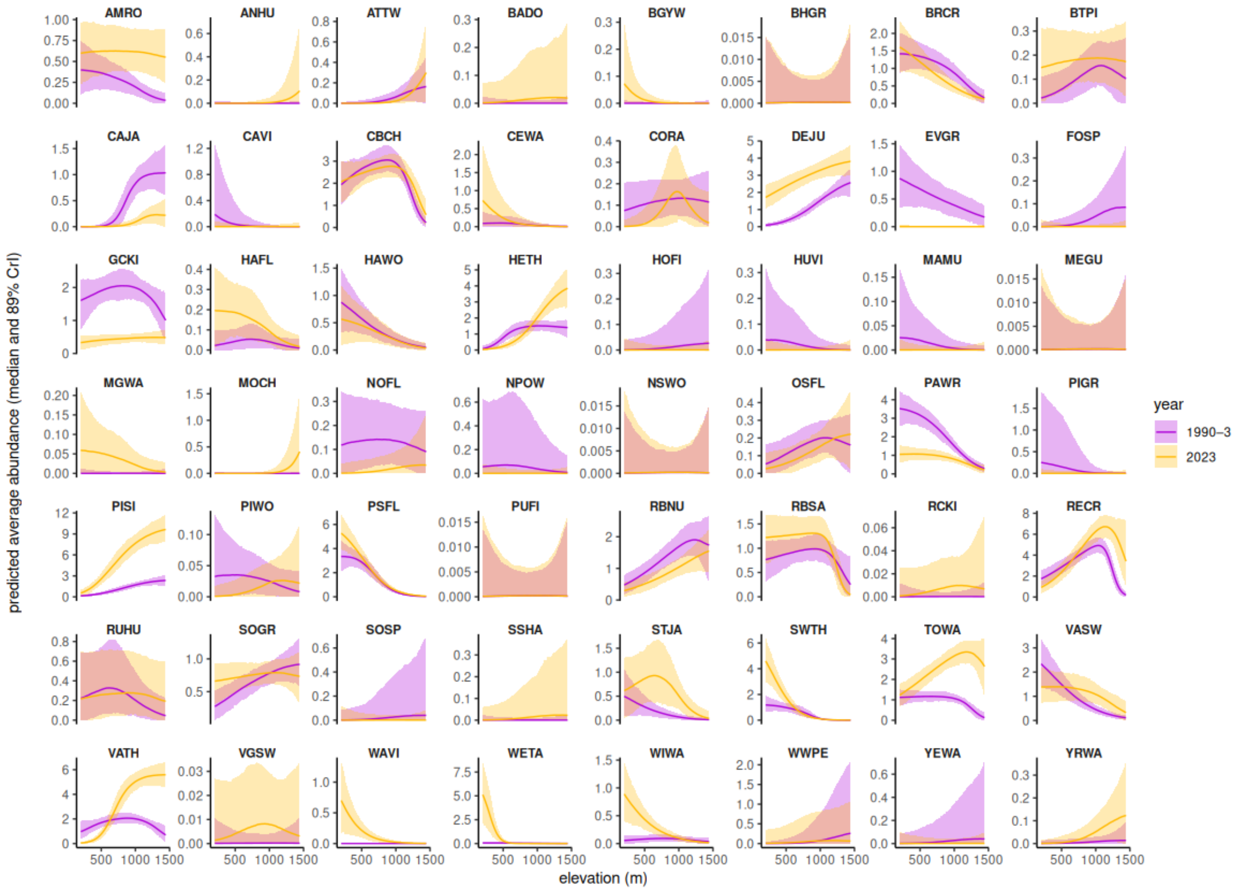

Figure S1. Modeled abundance distributions along the elevational gradient for both time periods for all species detected. Note that y-axis scales are different between species.

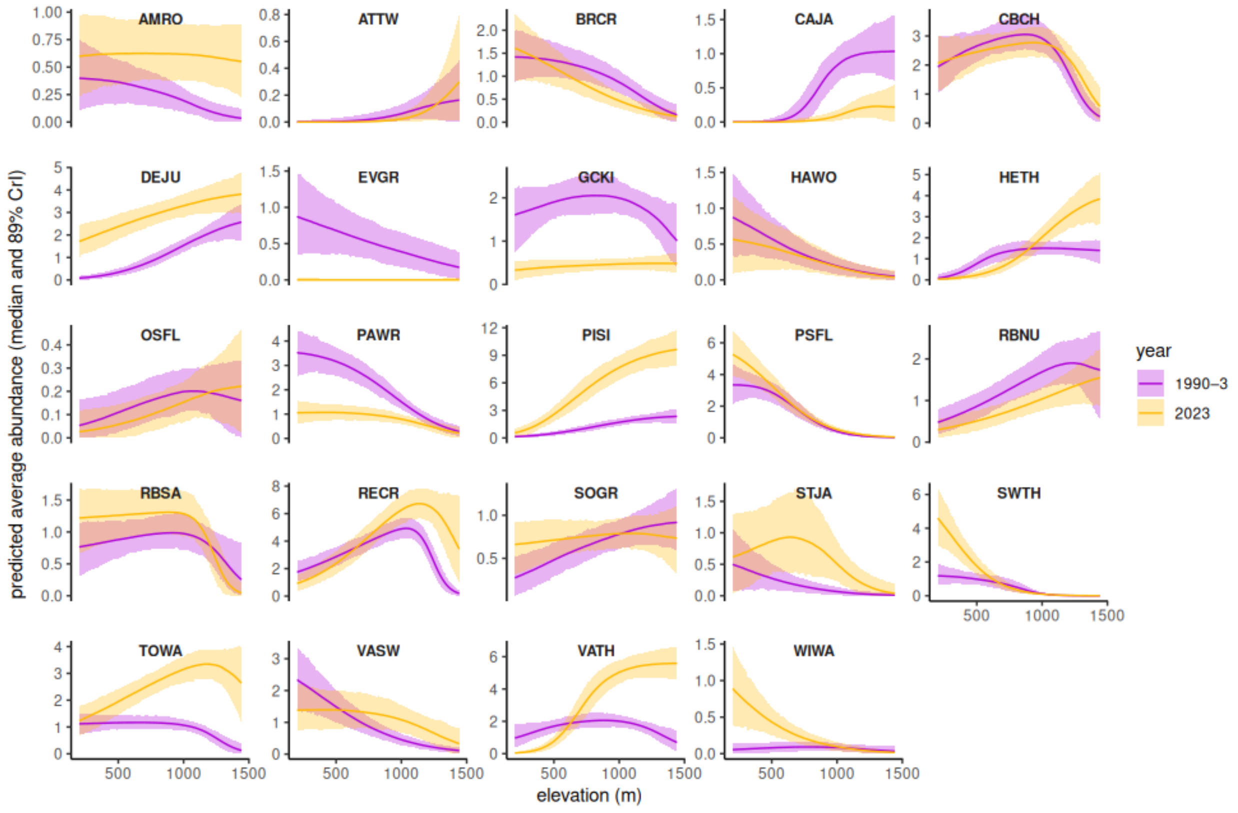

Figure S2. Modeled abundance distributions along the elevational gradient for both time periods for each of 24 common species that were analyzed. Note that y-axis scales are different between species.

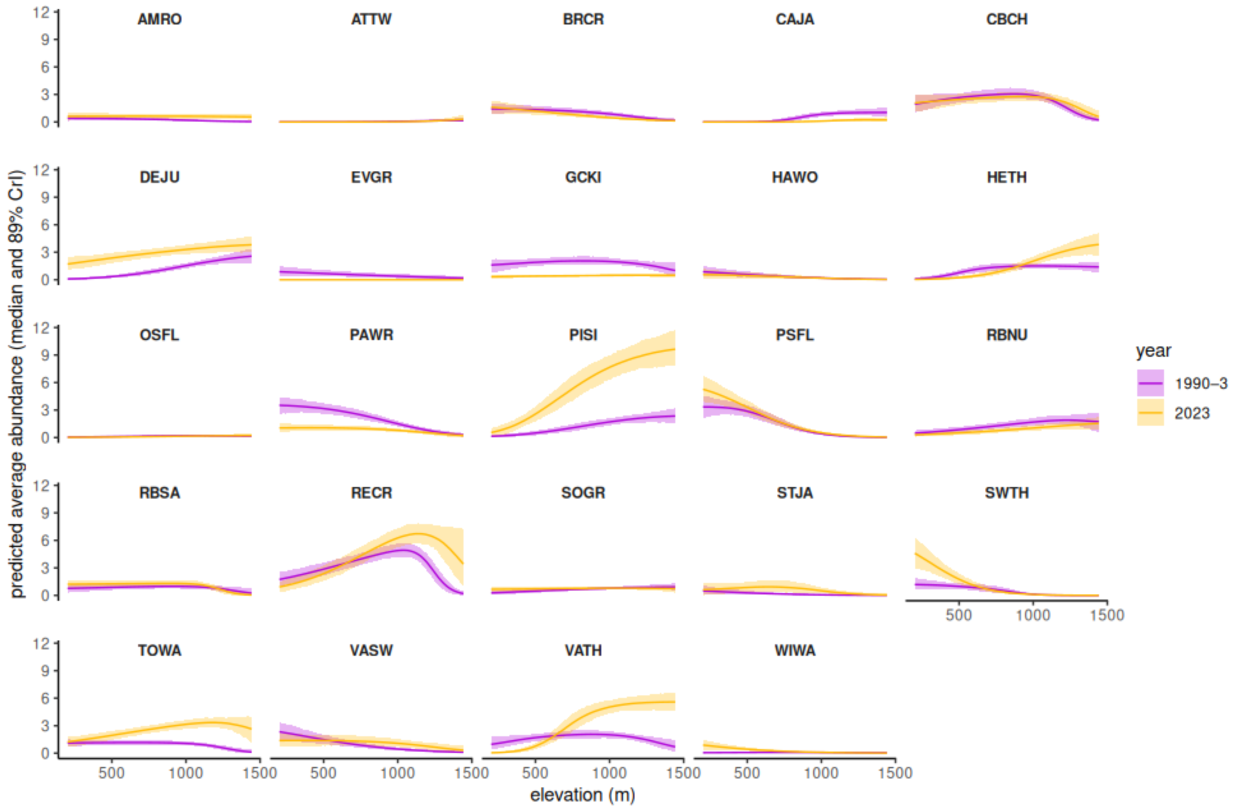

Figure S3: Modeled abundance distributions along the elevational gradient for both time periods for 24 common species with a constant y-axis to show absolute differences in abundance across species.

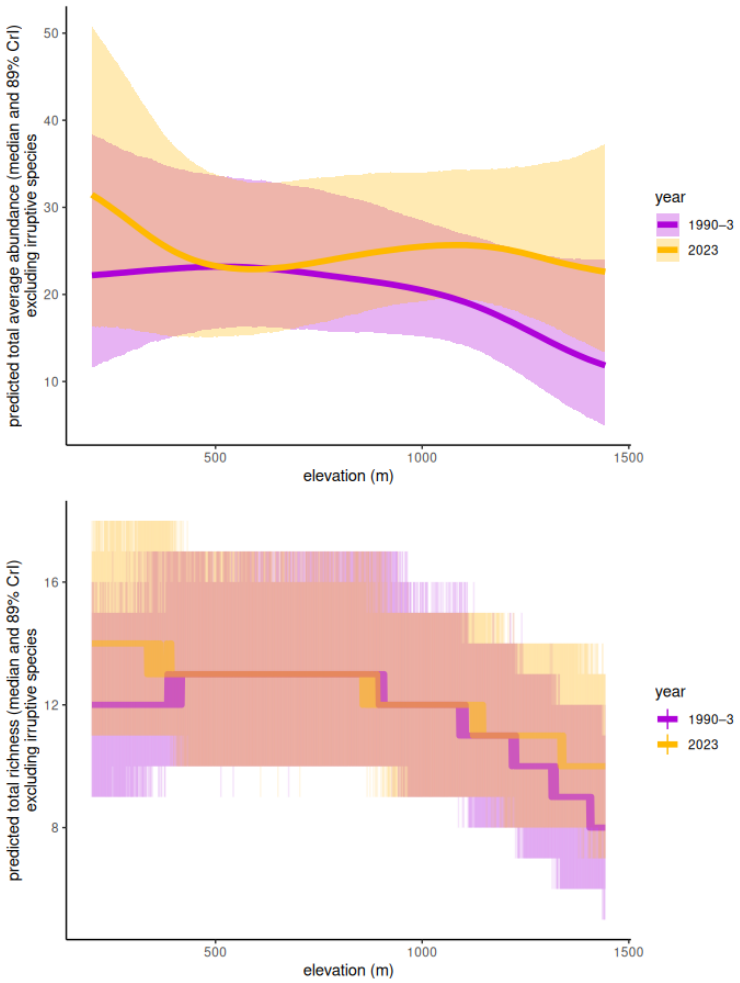

Figure S4 - The community-level metrics of abundance and species richness, with irruptive finch species (Evening Grosbeak, Red Crossbill, Pine Siskin) removed.
